# Comparison of implant placement at crestal and subcrestal levels in aesthetic zone: A finite element analysis

**DOI:** 10.1111/jopr.13930

**Published:** 2024-08-13

**Authors:** Taha Özer, Gülin Acar

**Affiliations:** ^1^ Department of Oral and Maxillofacial Surgery Hacettepe University Ankara Turkey

**Keywords:** alveolar bone loss, alveolar process, biomechanical phenomena, dental implants, finite element analysis, tensile stress

## Abstract

**Purpose:**

The success rate of the implant treatment, including aesthetics and long‐term survival, relies heavily on preserving crestal peri‐implant bone, as it determines the stability and long‐term outcomes. This study aimed to demonstrate the stress differences in the crestal bone resulting from dental implant placement at various depths relative to the crestal bone level using finite element analysis.

**Materials and Methods:**

Three study models were prepared for implant placement at the crestal bone level (CL), 1 mm depth (SL‐1), and 2 mm depth (SL‐2). Implants were placed in the maxillary central incisor region of each model, and 100 N vertical and oblique forces were applied. The von Mises, maximum principal (tensile), and minimum principal (compressive) stresses were evaluated.

**Results:**

The CL model exhibited the highest stresses on the implant, abutment, and abutment screws under vertical and oblique forces. For maximum principal stress in the crestal bone under vertical force, the SL‐2, SL‐1, and CL models recorded values of 6.56, 6.26, and 5.77 MPa, respectively. Under oblique forces, stress values for SL‐1, SL‐2, and CL were 25.3, 24.91, and 23.76 MPa, respectively. The CL model consistently exhibited the lowest crestal bone stress at all loads and the highest stress values on the implant and its components. Moreover, considering the yield strengths of the materials, no mechanical or physiological complications were noted.

**Conclusions:**

Placing the implant at the crestal level or subcrestally beyond the cortical layer could potentially reduce stress and minimize crestal bone loss. However, further studies are warranted for confirmation.

A single missing tooth, a commonly encountered clinical situation, can result from trauma, dental caries, or congenital causes. The replacement of such missing teeth in patients presenting to clinics for aesthetics and/or functional restoration has become one of the most urgent needs. Numerous treatment options are available, each with its own advantages and disadvantages.^1^ Dental implants, along with treatment alternatives including fixed and removable partial dentures, are considered the gold standard among these options owing to their advantages of long‐term predictability, and high aesthetic results, functionality, and success rates.^2^

With recent developments in the field of implantology, numerous researchers have studied the surface properties and geometrical design of implants. The crestal peri‐implant bone status is an essential criterion for determining the success of implants.^3^ Preservation of the crestal peri‐implant bone is crucial for the success rate of the treatment, including aesthetics and long‐term survival, as it determines the stability of the peri‐implant bone and soft tissues.^4^ However, crestal peri‐implant bone loss is assumed to occur over the years after implant placement, and its extent is unpredictable.^5^ The position of the implant is the main factor preventing future crestal bone loss. However, the ideal implant position according to the crestal level remains controversial.^6^ Although it has been pointed out that subcrestal placement of implants could result in less peri‐implant bone loss in the future, no literature data are available on the depth of this level. Furthermore, studies have examined features such as the microbial environment, implant surface and geometric properties, implant‐abutment connection types, and the condition of soft tissues.^6^ However, no study is available regarding the forces that may cause crestal bone loss and the distribution of the stresses on the bone caused by these forces.

Finite element analysis (FEA) is a numerical technique used to analyze and simulate physical phenomena. It relies on mathematical models to understand and measure a variety of physical phenomena, including structural and fluid behavior, mechanical stress, thermal transport, wave propagation, and the growth of biological cells.^7^ In the medical field, they are particularly useful for studying complex biomechanical systems that are difficult to study in vivo and in vitro. It is used to estimate the mechanical response of tissues under different stimulation conditions and to evaluate structural changes under both healthy and pathological conditions.^7^ Recently, numerous studies have been conducted on the application of FEA in oral implantology. Most research has focused on studying the mechanical behavior of implants during functional loads to evaluate the stress and strain fields in the implant components and bone at a macroscale.^8^, ^9^

The relationship between the implant level and the alveolar crest is essential for understanding crestal bone loss; however, no specific research has been conducted regarding this subject. The null hypothesis of this study is that as implants are placed deeper than the marginal bone level, the stress created by vertical and oblique forces on the bone decreases. Therefore, this study aimed to compare the stresses created in the maxilla by implants placed crestally and subcrestally at various levels under physiological forces in the treatment of maxillary single‐tooth deficiencies in the aesthetic region using the FEA method.

## MATERIALS AND METHODS

**FIGURE 1:**
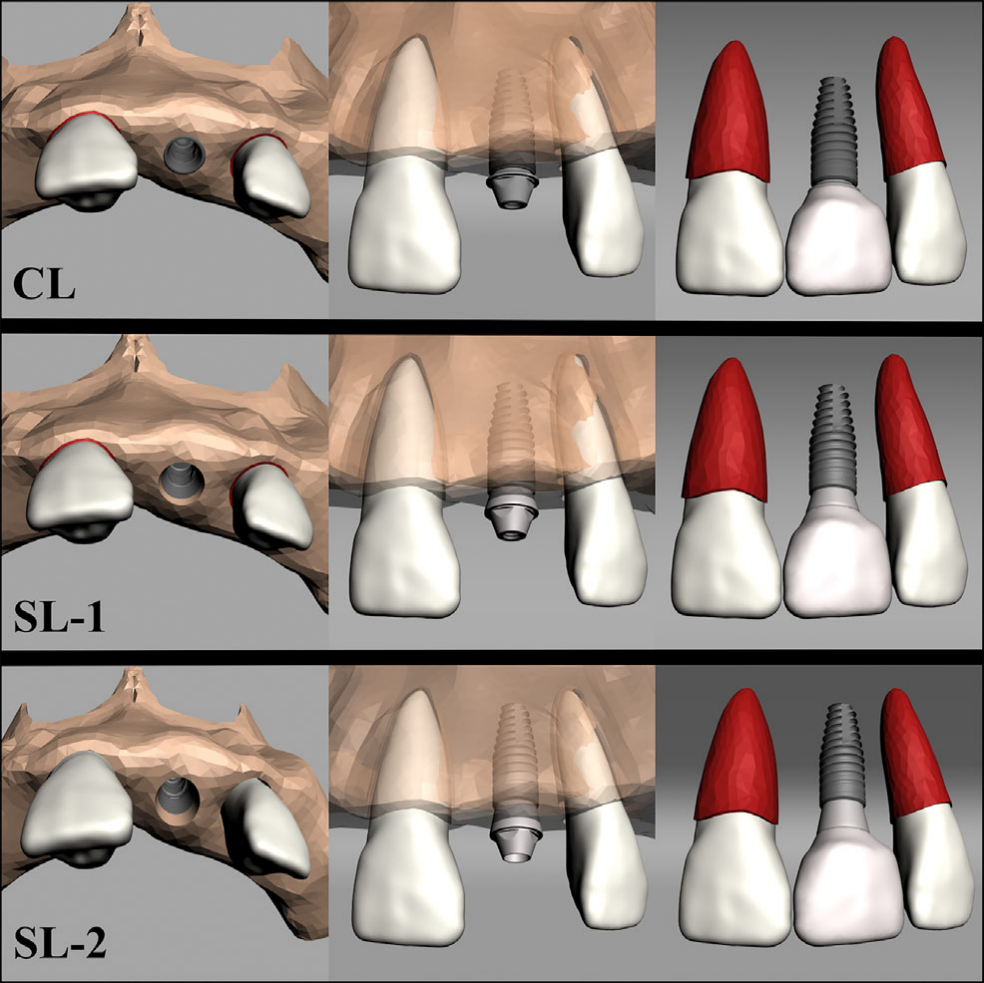
Implants positioned at three different depths according to the alveolar crest level. CL model: the implant is placed at the crestal level; SL‐1 model: the implant is positioned 1 mm below the crestal level; and SL‐2 model: the implant is positioned 2 mm below the crestal level.

**FIGURE 2:**
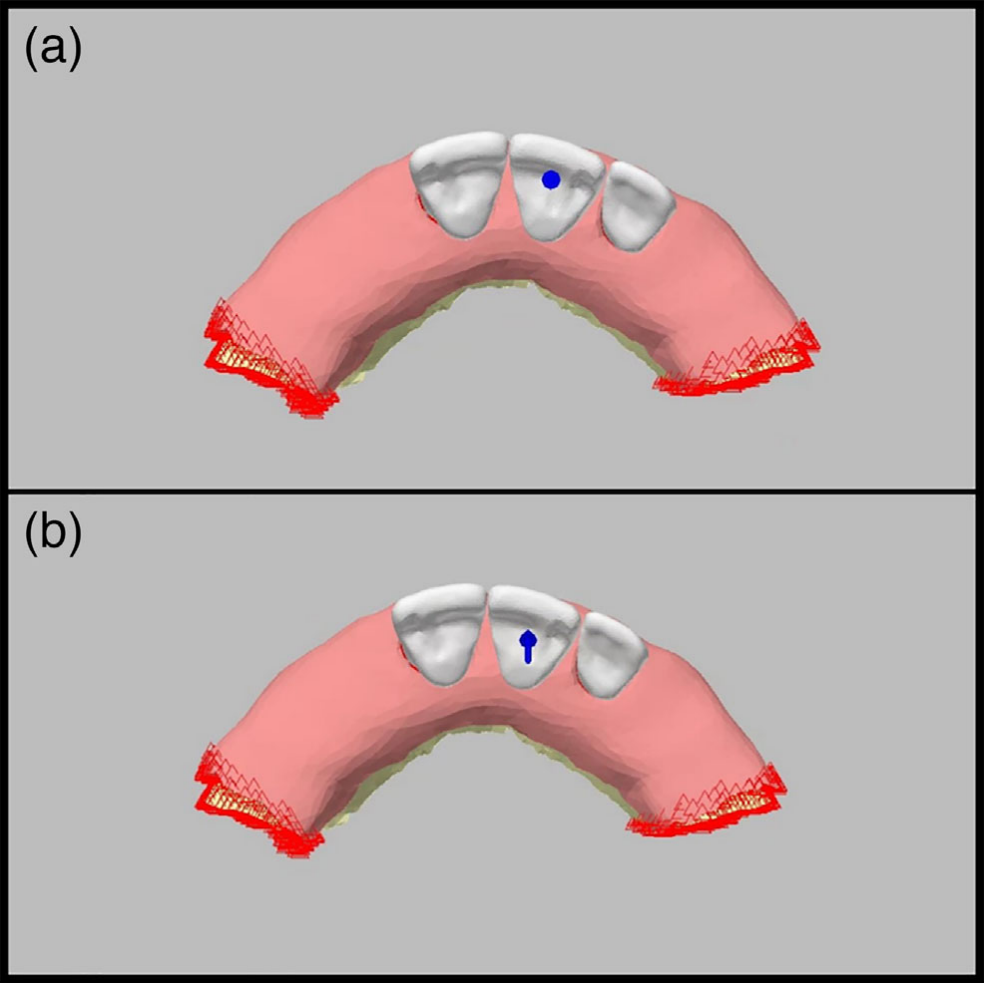
Boundary conditions with different loading types: (a) Vertical loading with 100 N; (b) Oblique loading with 100 N.

This study was designed to compare the stress values on the implants, implant parts, and bone under vertical and oblique forces with models with implants at the crestal level (C, 1 mm below the crest level, and 2 mm below the crest level to determine the ideal axial position of the implants in the bone. All groupsʼ implants were positioned at maxillary lateral tooth region. The implant was placed at the crestal level in Model CL. Further, the implants were positioned 1 and 2 mm below the crestal level in Models SL‐1 and SL‐2, respectively. This positional difference was eliminated by increasing the gingival height of the screw‐retained abutments, allowing restoration to start at the gingival level in all three models (Figure 1). A force of 100 N was applied perpendicularly and at 45° obliquely from the same point (Figure 2).

Rhinoceros 4.0, a three‐dimensional (3D) modeling software (3670 Woodland Park Ave N, Seattle, WA 98103 USA), VRMesh Studio (VirtualGrid Inc., Bellevue City, WA, USA), and Algor Fempro (ALGOR, Inc., 150 Beta Drive Pittsburgh, PA 15238‐2932 USA) analysis programs were utilized to arrange and homogenize a 3D mesh structure, create a 3D solid model, and perform finite element stress analysis. A 3D upper jaw model was created based on a computed tomographic scan of a normal human maxilla with missing central incisors. The model was generated using Able (V4.0) software STL file format and transferred to the VRMesh (V7.1) software. Using this software, surface data from STL format was obtained using available triangulated mesh data obtained from the 3D model. Finally, the models were transferred to Algor Fempro software (Algor Inc., USA) in STL format to prepare them for analysis. The alveolar bone height was set at 19 mm from the top of the alveolar crest to the base of the nose, and the alveolar bone thickness was set at 8 mm. The cortical bone thickness was set at 1.5 mm, and the gingival thickness was determined at 1.5 mm. A Straumann bone‐level tapered titanium implant (Waldenburg, Switzerland) with a length of 10 mm and a diameter of 4 mm was used. Screw‐retained 0° titanium abutments (Waldenburg, Switzerland) with 1.5, 2.5, and 3.5 mm gingival heights were selected as abutments and adapted to the scenarios.

All layers of the three models were composed of homogeneous and isotropic linear elastic materials, and each of the structures was assigned material values (Young's modulus (*E*) and Poisson ratio (*μ*)) describing their physical properties based on published data (Table 1).

**TABLE 1:** Material properties used in FEA analysis.

Material	Young's modulus (GPa)	Poisson's ratio (*μ*)
Titanium abutment and implant	115	0.35
CoCr alloy	218	0.33
Feldspathic porcelain	82.8	0.35
Cortical bone	13.7	0.3
Spongious bone—D3	1.6	0.3
Mucosa	1	0.37
Tooth	18.6	0.31
Periodontal ligament	0.0689	0.45

Abbreviations: CoCr, cobalt–chromium; FEA, finite element analysis.

In the prepared models, osseointegration of the implant was considered 100%, and screw‐retained abutments and occlusal screws were used as separate units. A cobalt–chromium (CoCr) alloy was used for the construction of the superstructure. Although the loading conditions and superstructures were the same in each model, the implants were placed in three different positions according to the alveolar crest level. Modeling and solution procedures were performed using the Algor Fempro software.

After the models used in the study are prepared and geometric models are created with the help of VR Mesh, point data can be accessed. The data obtained in this study are the maximum values occurring around the material and were evaluated among themselves.

Vertical or oblique loads cause compression or tension in the crestal cortical bone around the implant neck. Therefore, the analysis of the tensile and compressive stresses in these regions is critical. The von Mises stress is used to evaluate the stresses occurring within ductile materials such as metallic implants. The types of stress evaluated in this study were Von Mises, maximum principal (tensile), and minimum principal (compressive) stresses.

## RESULTS

The values obtained in the FEA studies were unrepeatable and deviation‐free mathematical calculations. Therefore, no statistical analyses were performed. The results were carefully analyzed and interpreted. The von Mises, maximum principal, and minimum principal stress values measured for all three models are listed separately.

**FIGURE 3:**
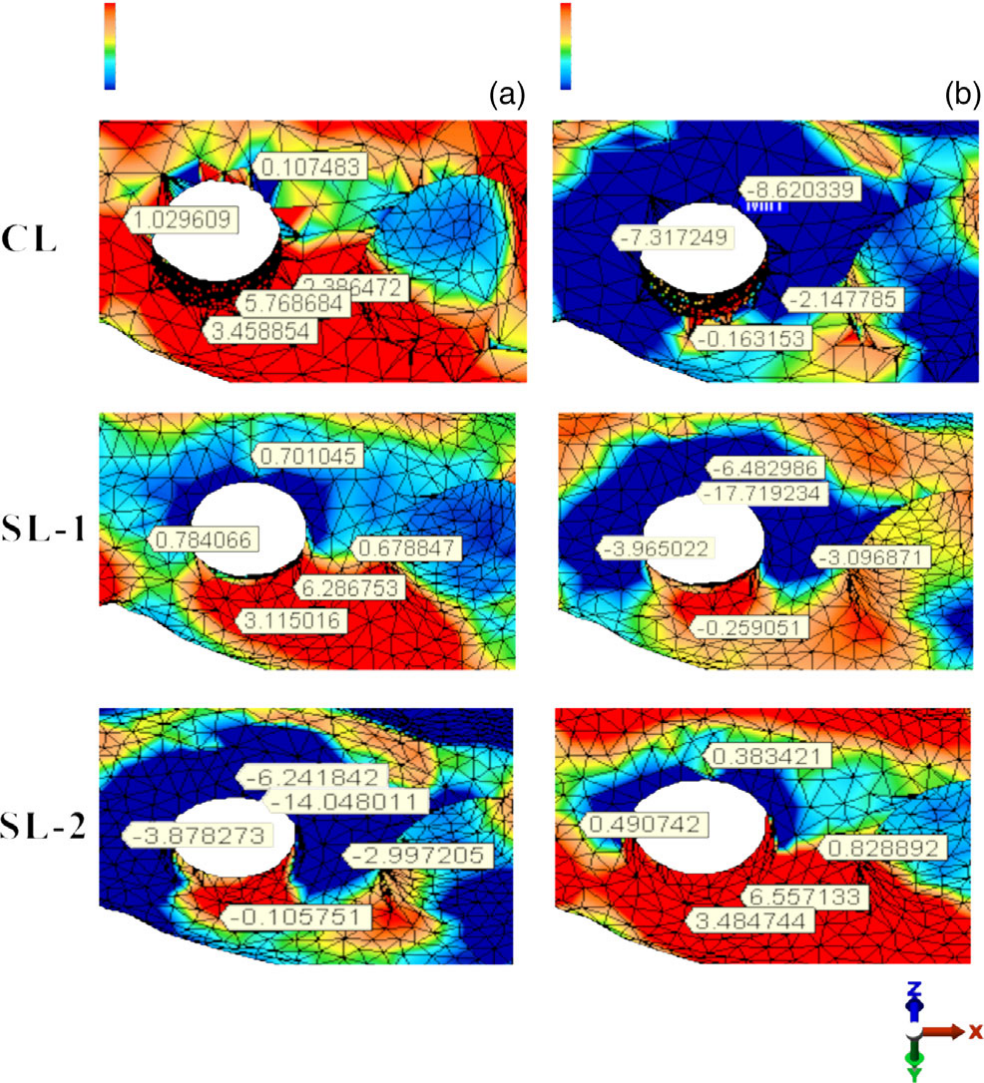
Stress distribution on the peri‐implant cortical bone in the models: (a) Tensile stresses with vertical loading; (b) Compressive stresses with vertical loading.

The compressive stress distributed to the crestal cortical bone under 100 N vertical load was measured as −15.95, −17.72, and −14.05 MPa for Models CL, SL‐1, and SL‐2, respectively. The tensile stresses distributed under the same conditions were measured at 5.77, 6.29, and 6.56 MPa, respectively (Figure 3, Table 2).

**TABLE 2:** Maximum compressive and tensile stresses in the peri‐implant cortical bone at different loading conditions.

		Model CL	Model SL‐1	Model SL‐2
**Vertical** (100 N)	Tensile	5.77	6.29	6.56
	Compressive	−15.95	−17.72	−14.05
**Oblique** (100 N)	Tensile	23.76	25.30	24.91
	Compressive	−29.89	−33.90	−27.02

Model CL, implant placement at the crestal bone level; Model SL‐1, implant placement at 1 mm below the crestal bone level; Model SL‐2, implant placement at 2 mm below the crestal bone level.

**FIGURE 4:**
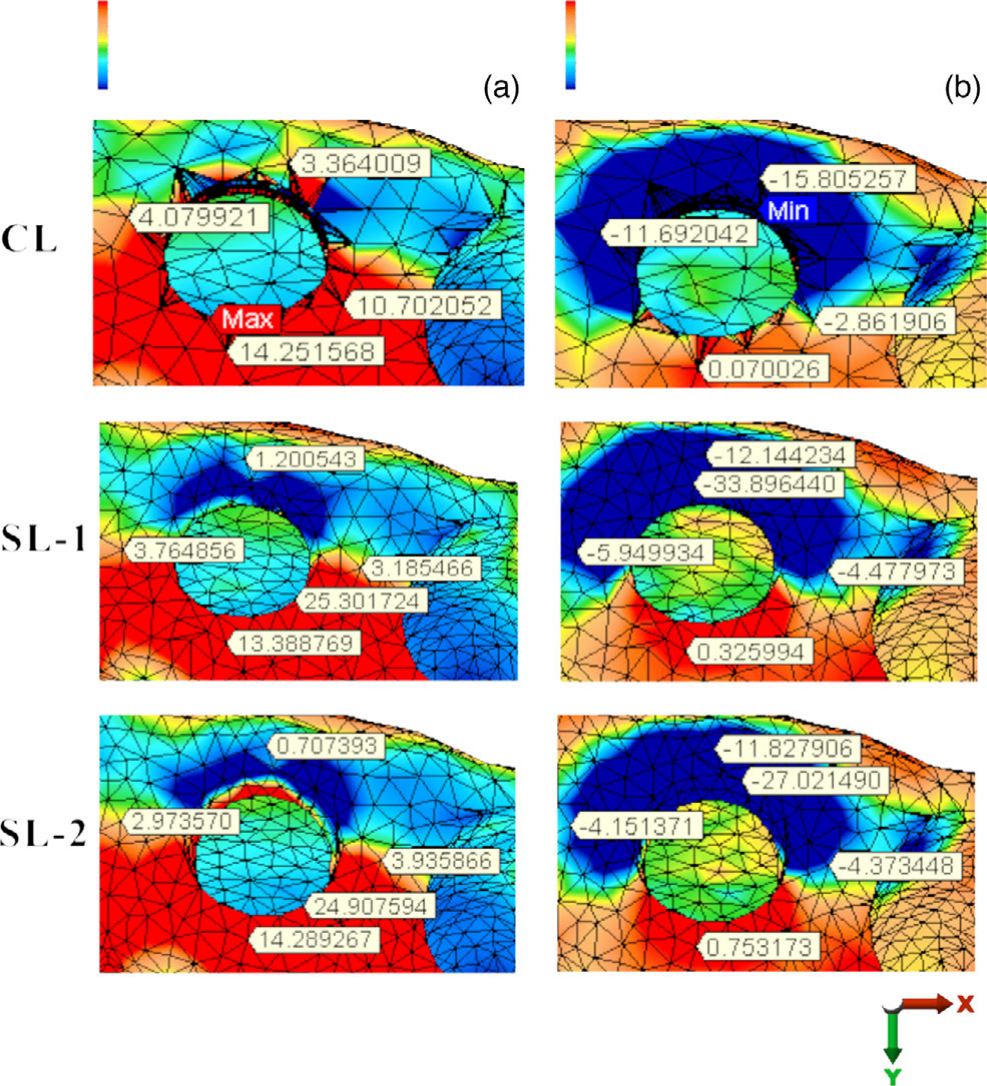
Stress distribution on the peri‐implant cortical bone in the models: (a) Tensile stresses with oblique loading; (b) Compressive stresses with oblique loading.

The compressive stress distributed at the crestal cortical bone under 100 N oblique load was measured as −29.89, −33.90, and −27.02 MPa for Models CL, SL‐1, and SL‐2, respectively. The tensile stress distributions under the same conditions were 23.76, 25.30, and 24.91 MPa, respectively (Figure 4, Table 2). All the stress distribution areas and values are shown in Figures 3 and 4 and Table 2.

The stress distributions on the abutments, implants, and screws were concentrated at the neck of the abutments and implants and the head of the abutment and occlusal screws in all three models under vertical and oblique loads. The magnitude of stresses was concentrated on the implant, abutment, and abutment screw in Model CL.

The stresses distributed on the implant under 100 N vertical load were found to be 50.30, 34.40, and 43.10 MPa in Models CL, SL‐1, and SL‐2, respectively. Under an oblique load of 100 N, these values were 112.79, 81.69, and 95.34 MPa, respectively.

The stresses distributed on the abutment under 100 N vertical load were found to be 88.71, 46.43, and 66.37 MPa in the CL, SL‐1, and SL‐2 models, respectively. Under an oblique load of 100 N, these were 168.21, 126.55, and 129.69 MPa, respectively.

The stresses distributed on the abutment screw under 100 N vertical load were found to be 73.02, 66.51, and 65.46 MPa in Models CL, SL‐1, and SL‐2 models, respectively. Under an oblique load of 100 N, these values were 116.90, 63.77, and 77.14 MPa, respectively.

**FIGURE 5:**
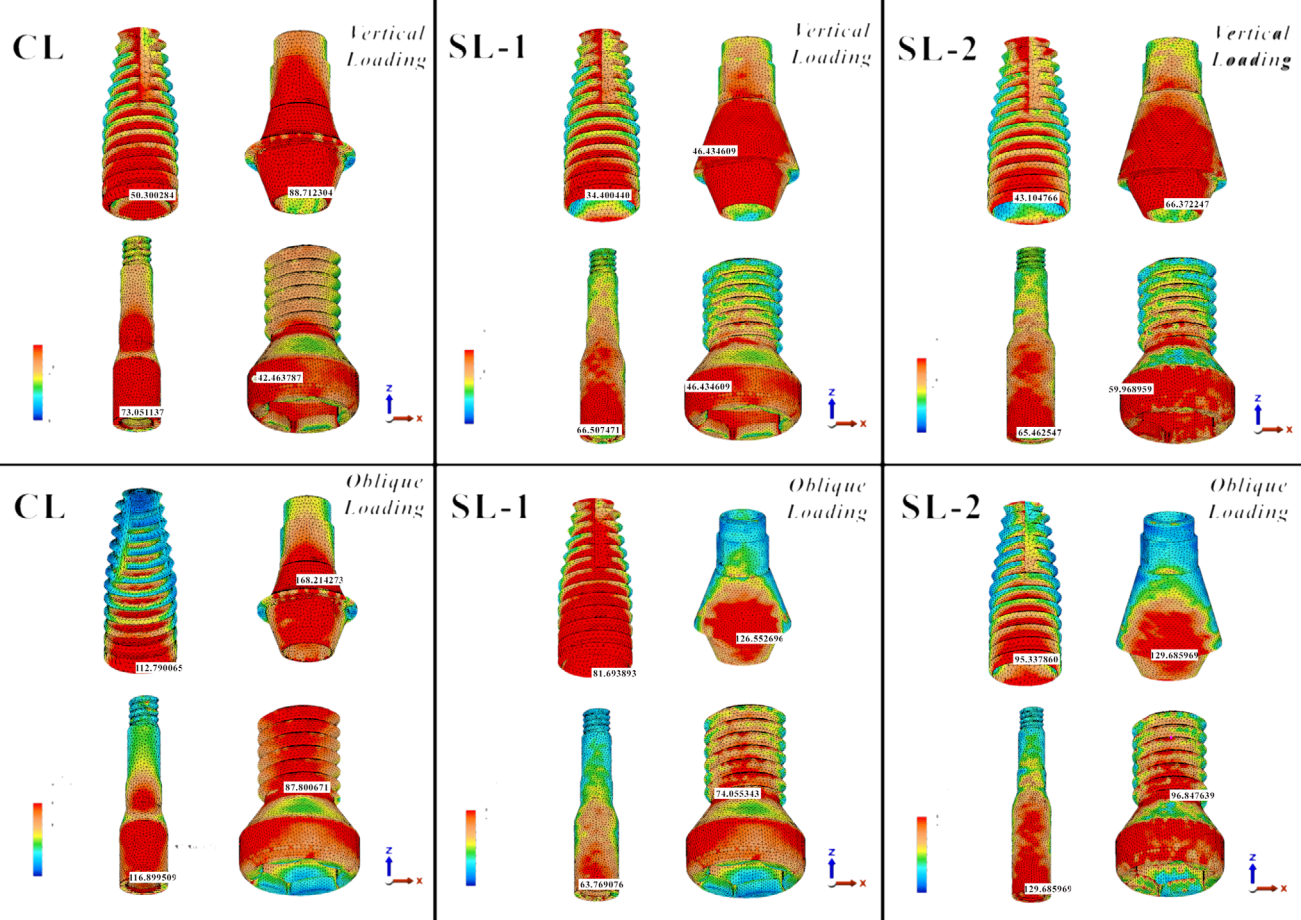
The distribution of von Mises stresses around the implant and implant components.

The stresses distributed on the occlusal screw under a 100 N vertical load were found to be 57.94, 57.43, and 59.97 MPa in the CL, SL‐1, and SL‐2 models, respectively. Under an oblique load of 100 N, these values were 87.80, 74.05, and 96.85 MPa, respectively. The stress distribution areas and values are shown in Figure 5 and Table 3.

**TABLE 3:** Maximum von Mises stress in different components of implants under different loading conditions.

		Model CL	Model SL‐1	Model SL‐2
**Vertical** (100 N)	Abutment	88.71	46.43	66.37
	Abutment screw	73.02	66.51	65.46
	Occlusal screw	57.94	57.43	59.97
	Implant	50.30	34.40	43.10
**Oblique** (100 N)	Abutment	168.21	126.55	129.69
	Abutment screw	116.90	63.77	77.14
	Occlusal screw	87.80	74.05	96.85
	Implant	112.79	81.69	95.34

Model CL, implant placement at the crestal bone level; Model SL‐1, implant placement at 1 mm below the crestal bone level; Model SL‐2, implant placement at 2 mm below the crestal bone level.

## DISCUSSION

Similar to other bones of the human skeleton, the maxilla and mandible show specific trends toward progressive bone loss over time. Therefore, studies on the resorption tendency of the peri‐implant bone have become increasingly sought‐after since it was proposed that dental implant‐supported fixed prostheses could be used for a lifetime.^10^ Recently, the causes of minimal bone loss from the moment dental implants are placed in the bone, especially during the first few months, have been investigated. According to these studies, vertical placement of the implant based on the bone level is considered the primary cause of bone loss.^6^ This study aimed to determine the stresses on the peri‐implant crestal bone caused by implants placed at various depths with respect to the crestal bone level, which is the most significant factor causing bone loss in the peri‐implant region, using FEA.

Determinants of the clinical success of dental implants include immobility of the implants, minimal crestal bone loss, and the absence of permanent morbidity.^11^ Peri‐implant crestal bone resorption between 0.9 and 1.6 mm in the first year and between 0.05 and 0.13 mm per year in the following years was considered normal in previous studies.^12^ Using a simple mathematical calculation based on these data, this resorption process leads to a bone loss of approximately 8 mm in 50 years. This results in a highly detrimental outcome for young adults, particularly concerning dental implications.

Every implant is expected to cause bone loss from the moment it is placed in the bone, which may lead to implant exposure, aesthetic problems, and peri‐implant pathologies. Considering this, it may be preferable to position the implant deeper than at the alveolar crest to avoid these potential disadvantages. A clinical study by Salina et al. compared the crestal bone loss around implants placed 0.5 and 1.5 mm subcrestally and demonstrated that the bone loss around implants placed at a depth of 1.5 mm was significantly reduced even after 3 years of implant loading. However, they did not find a significant difference between the groups in the aesthetic scoring performed using the pink aesthetic score and emphasized that this bone loss was insignificant regarding aesthetics.^13^ A similar study by Koutouzis et al. compared the crestal bone level with the level of the implant‐abutment connection, with three separate study groups consisting of implants placed at the crestal level, 1 mm subcrestally, and 2 mm subcrestally.^14^ At the end of 1 year, the highest resorption was observed in the group of implants placed at a depth of 2 mm, whereas the lowest resorption was observed in the group of implants placed at the crestal level. However, they showed that the resorption of implants at the crestal level, although minimal, exposed the implant‐abutment connection surface. After 12 months, only 35% of the bone‐level implants showed the presence of bone at the implant‐abutment connection surface, whereas this rate was 90% in the other two groups, and the crestal bone was above the bone‐implant contact point.^14^

In studies investigating the main causes of crestal bone resorption with this multifactorial etiology, it is necessary to consider several factors concurrently, including platform switching, type of connection, soft tissue characteristics, and biological width, although the most significant factor appears to be the vertical position of the implant relative to the crest level.

In the platform switching concept, the implant/abutment connection is positioned toward the center of the implant to separate bacterial filtration from the crestal bone.^15^ In previous studies, subcrestally placed platform switching implants showed greater crestal bone loss, which was attributed to deeper pocket formation.^3^, ^16^ Some other studies have argued in favor of the platform‐switching concept in subcrestally placed implants, showing that it causes less crestal bone loss.^15^, ^17^, ^18^ Therefore, the relevance of platform switching in bone resorption remains controversial.

Numerous clinical studies have suggested that the Morse taper connection with a subcrestal implant may be suitable for preventing bone loss and is the most effective implant‐abutment connection for bacterial sealing and prosthesis stability. However, there are no adequate data in the literature on the relationship between internal or external implant‐abutment connection type and crestal loss.^6^, ^15^

The formation of biological width around the implant is one of the most influential factors in bone remodeling that starts from the moment the implant is opened to the oral environment, which explains why resorption is much higher in the first year. In multiple studies, implants placed at the crestal level with a thin gingival biotype showed greater bone loss than those placed subcrestally. The general opinion is to leave a gap of at least 3 mm in total for the formation of biological width and to place the implant 1 mm subcrestally in patients with a mucosal thickness of 2 mm.^18^, ^19^, ^20^

Another significant factor in the resorption of the crestal bone around the implant, which is a multifactorial event, is the stress that occurs on an implant under load and is transmitted to the underlying alveolar bone. The relationship between the changes in the resorption of the crestal bone and the vertical positioning of the implant relative to the crestal bone and the changes in the stresses transmitted to the bone has not been the subject of any previous study. In this study, the maxillary anterior region, which has the highest incidence of aesthetic complications due to crestal bone loss, was selected. Considering the structure of the maxilla, the bone density was created as D3 in the model, and the cortical bone thickness was assumed to be 1.5 mm.^21^ Considering other studies, analyses were performed by applying vertical and oblique forces of 100 N.^22^ Failure occurred when the stress values measured on the cortical bone exceeded the yield strength of the cortical bone. According to a study by Baggi et al., the absolute values of the highest tensile and compressive strengths of cortical bone were obtained as 100–130 MPa and 170–190 MPa, respectively.^23^ The highest tensile and compressive stress values obtained in this study were 25.3 and 33.9 MPa, respectively. These values show that the stresses transmitted to the bone in all the groups and directions of forces were physiological.

The stresses on the implant, abutment, abutment screw, and occlusal screw were not sufficiently large to cause mechanical complications, considering the yield strength of the materials. Among the components, the highest stress was observed in the CL model.

The highest stress transmitted to the crestal bone under both vertical and oblique forces was observed in the SL‐1 group. A cortical bone thickness of 1.5 mm can be considered a contributing factor. Therefore, it can be concluded that the neck of the implant embedded in the cortical layer of the bone creates more stress in the crestal bone. In the SL‐1 model, the neck of the implant was located within the cortical layer, whereas in the SL‐2 model, the neck of the implant crossed the cortical layer and terminated in the trabecular bone, creating lower stress in the crestal bone.

For the tensile‐type stresses, the model with the lowest stress was CL, whereas for the compressive‐type stresses, the model with the lowest stress was SL‐2. Based on these results, if the vertical position of the implant with respect to the crestal bone is to be negatively positioned, it must pass through the cortical layer or leave it at the crestal level. Thus, the stress on the crestal bone can be minimized. However, as mentioned before, it should be noted that stress is not the only influencing factor in peri‐implant bone resorption, which is a multifactorial process, and it should be evaluated together with other factors affecting resorption.

As in all other FEA studies, the differences between the clinical cases and models used in the analysis are one of the limitations of this study. Despite the ability of FEA to minimize errors, it is not necessarily possible to directly simulate a clinical scenario.^24^

## CONCLUSIONS

This study aimed to determine the optimum crestal bone‐implant level to minimize crestal bone loss in the anterior maxillary region, which can lead to severe aesthetic complications. Considering these findings, it is recommended to leave the implant at the level of the crest, which induces the lowest stress, or to position the implant at a depth that crosses the cortical layer of the bone. However, further clinical studies are required to confirm these findings.

## CONFLICT OF INTEREST STATEMENT

The authors have no conflict of interest related to the study.

## Data Availability

The datasets used and/or analyzed during the current study are available from the corresponding author upon reasonable request.
